# Oligodendrocyte, Astrocyte, and Microglia Crosstalk in Myelin Development, Damage, and Repair

**DOI:** 10.3389/fcell.2016.00071

**Published:** 2016-06-28

**Authors:** Helena S. Domingues, Camila C. Portugal, Renato Socodato, João B. Relvas

**Affiliations:** ^1^Glial Cell Biology Group, Instituto de Biologia Molecular e Celular, Universidade do PortoPorto, Portugal; ^2^Glial Cell Biology Group, Instituto de Investigação e Inovação em Saúde (I3S), Universidade do PortoPorto, Portugal

**Keywords:** oligodendrocyte, astrocyte, microglia, multiple sclerosis (MS), experimental autoimmune encephalomyelitis (EAE), myelination, demyelination, remyelination

## Abstract

Oligodendrocytes are the myelinating glia of the central nervous system. Myelination of axons allows rapid saltatory conduction of nerve impulses and contributes to axonal integrity. Devastating neurological deficits caused by demyelinating diseases, such as multiple sclerosis, illustrate well the importance of the process. In this review, we focus on the positive and negative interactions between oligodendrocytes, astrocytes, and microglia during developmental myelination and remyelination. Even though many lines of evidence support a crucial role for glia crosstalk during these processes, the nature of such interactions is often neglected when designing therapeutics for repair of demyelinated lesions. Understanding the cellular and molecular mechanisms underlying glial cell communication and how they influence oligodendrocyte differentiation and myelination is fundamental to uncover novel therapeutic strategies for myelin repair.

## Introduction

Glial cells, neuroglia, or simply glia, in the adult mammalian central nervous system (CNS) comprise astrocytes, oligodendrocytes, and microglia. Collectively, they are by far the most abundant cells in the nervous system. The term glia, derived from the Greek word meaning glue, reflects the nineteenth-century view of Rudolph Virchow that these cells had the function to hold the nervous system together (Virchow, 1856). Today, we know that glia play many other roles, such as modulation of homeostatic functions, myelination, synaptic function, nerve signal propagation and responses to neural injury (for extended information please see reviews Herculano-Houzel, 2014; Zuchero and Barres, 2015).

Astrocytes have star-shape morphology and are the most abundant CNS glial cell type. They play essential functions in blood brain barrier maintenance, neuronal survival, and in synapse formation, strength, and turnover (Barres, 2008). First characterized by del Río-Hortega (1928), oligodendrocytes are the myelinating glia of the CNS (Nave and Werner, 2014) and their myelin sheaths enwrap axons to allow fast saltatory conduction of action potentials. They also provide axonal metabolic support (Funfschilling et al., 2012) and contribute for neuroplasticity (Mckenzie et al., 2014). While oligodendrocytes and astrocytes originate from a common lineage of neural progenitor cells within the neuroectoderm, microglia are the main innate immune cells of the CNS and arise from hematopoietic stem cells in the yolk sac during early embryogenesis that populate the central nervous system. Being ontogenetically different from other tissue-macrophages they have longevity and capacity for self-renewal (Chan et al., 2007; Prinz and Priller, 2014).

The roles of glial cells in health and in disease have been partially neglected because many basic aspects of their physiology and pathophysiology are still not completely understood. However, it is becoming more evident that glia-glia crosstalk plays several important roles in brain function during development and disease. This review discusses how in their active interplay, astrocytes and microglia can modulate oligodendrocyte homeostasis during myelination, demyelination and remyelination.

## Glial cell interactions in CNS (re)myelination and demyelination

### Oligodendrocyte differentiation in health and disease

#### Oligodendrocyte differentiation in developmental myelination

In the CNS, myelination is carried by oligodendrocytes. The myelin sheath is a modified and extended glial plasma membrane that enwraps around the axons enabling fast saltatory nerve conduction and axon integrity (Nave and Werner, 2014). Demyelination, the process or state resulting from the loss or destruction of myelin, is a hallmark of numerous diseases, such as multiple sclerosis (MS), contusion type spinal cord injury (SCI), and stroke. Oligodendrocytes derive from oligodendrocyte progenitor cells (OPC), which hold the capacity to proliferate, migrate, and differentiate into myelinating oligodendrocytes. Demyelination is often followed by remyelination, the default spontaneous process by which new OPC are recruited to differentiate into myelinating oligodendrocytes and the myelin sheaths are restored to axons, protecting them from degeneration. Ideally, remyelination should recapitulate developmental myelination. However, the inflammatory and activated milieu surrounding the demyelinated lesions compromises and limits the efficacy of the remyelination process (Franklin and Goldman, 2015).

**Figure 1:**
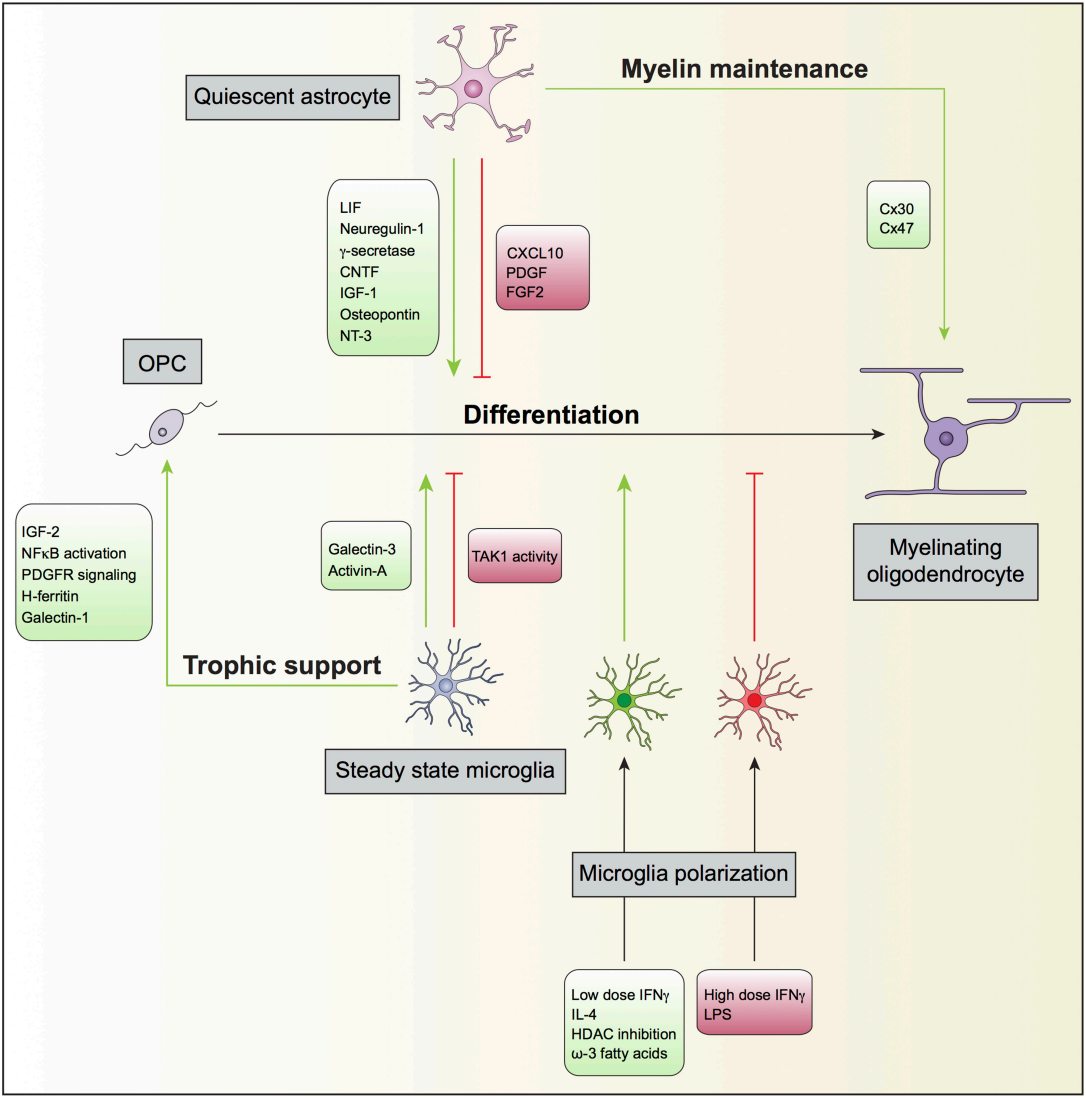
**Oligodendrocyte, astrocyte, and microglia crosstalk during developmental myelination**. In the non-diseased or non-insulted CNS, both quiescent astrocytes and steady state microglia may potentiate (green) or prevent (red) the differentiation of oligodendrocyte progenitor cells (OPC) into mature myelinating oligodendrocytes. Quiescent astrocytes can also support myelin maintenance, which will consequently enhance myelin production by myelinating oligodendrocytes. Steady state microglia might additionally contribute to oligodendrocyte differentiation by providing trophic support to OPC. Furthermore, microglia can modulate OPC differentiation directly after polarization by exogenous ligands. For instance, while the inflammagen LPS polarize microglia to prevent (red), the anti-inflammatory cytokine IL-4 polarize microglia to promote (green) OPC differentiation.

OPC are highly proliferative, motile and bipolar cells expressing high levels of the gangliosides recognized by the A2B5 antibody, the receptor of PDGF alpha (PDGFαR), and the NG2 proteoglycan. OPC specification and differentiation is regulated by transcription factors such as Olig1, Olig2, Mash, Myt1, Nkx2.2, and Sox10 and their differentiation along the oligodendroglial lineage, can be regulated by molecules such as IGF-1, FGF2, CNTF, and thyroid hormone T3 (see reviews from Zuchero and Barres, 2013; Mitew et al., 2014; Figure 1).

There are at least two identified sources of OPC in the adult brain: the progenitors from the subventricular zone (SVZ) (Menn et al., 2006) and the NG2 and PDGFαR positive OPC, believed to be homogeneously distributed within the CNS (Nishiyama et al., 1996; Watanabe et al., 2002; Rivers et al., 2008; Richardson et al., 2011). These cells account for 5–8% of all cells in the CNS (Levine et al., 2001) and exist throughout regions such as the optic nerve (Shi et al., 1998), motor cortex, corpus callosum (Clarke et al., 2012), and cerebellum (Levine et al., 1993), providing a substantial source of new oligodendrocytes and, thus, a potential reservoir for remyelination. Adult OPC are oligodendrocyte precursors with restricted lineage potential, generating myelinating oligodendrocytes, but not astrocytes or neurons, not even during disease (Kang et al., 2010). Interestingly, these cells are constantly proliferating in the CNS to maintain their homeostatic cell density (Hughes et al., 2013), though at slower rate than during development or injury (Shi et al., 1998; McTigue et al., 2001). Oligodendrocytes are generated continuously in the healthy adult brain. In human brain, although the oligodendrocyte turnover is very stable, with an annual change of 0.3% (Yeung et al., 2014), increasing evidence shows that myelin is produced and remodeled throughout life, not just during childhood and adolescence. Actually, it was shown that inhibiting the formation of new oligodendrocytes during adulthood, without compromising pre-existing oligodendrocytes and myelin, prevented mice from learning new motor skills (Mckenzie et al., 2014), suggesting that the formation of new myelinating oligodendrocytes during adult life is an important mechanism for neuroplasticity. Moreover, Birey and colleagues describe a new role for NG2 glial cells in the regulation of CNS homeostasis, other than their myelinating potential. The depletion of NG2 glia in the prefrontal cortex of the adult mouse brain caused deficits in excitatory glutamatergic neurotransmission and astrocytic extracellular glutamate uptake and induced depressive-like behaviors (Birey et al., 2015). Finally, Hughes and colleagues showed that NG2 positive cells also participate in the formation and resolution of the glial scar, suggesting that these progenitor cells are also able to detect CNS injury and promote tissue repair (Hughes et al., 2013).

OPC differentiation into a myelinating oligodendrocyte is characterized by a rapid increase in morphological complexity followed by expansion of uncompacted myelin membrane. Such profound morphological changes demand dynamic cytoskeleton rearrangements of microtubules and actomyosin (F-actin) (Bauer et al., 2009; Snaidero and Simons, 2014). In the past years, we and others have contributed to the identification and understanding of the role of regulatory proteins that govern the cytoskeletal function during developmental myelination in the CNS, namely the β1 integrin, the Rho GTPases Rac1 and Cdc42 and the extracellular matrix (ECM) proteins fibronectin and laminin (Relvas et al., 2001; Benninger et al., 2006; Thurnherr et al., 2006; Lourenco et al., 2016).

#### Oligodendrocyte differentiation after myelin damage

Following injury, and myelin damage, NG2/PDGFαR-expressing adult progenitors differentiate into oligodendrocytes capable of remyelinating axons (Zawadzka et al., 2010) and restoring nearly normal nerve conduction. However, over the course of MS changes in the microenvironment of the injured nervous system cause OPC to gradually loose the ability to respond to myelin damage limiting their remyelination capacity (Kipp et al., 2012). It is thought that for remyelination to occur, OPC need to be “activated.” These progenitor cells become responsive to mitogens, growth factors, chemokines and cytokines, which enhance their proliferation and mobilization to the demyelinated area, and increase the expression of genes associated with oligodendroglial differentiation (Redwine and Armstrong, 1998; Di Bello et al., 1999; Fancy et al., 2004; Moyon et al., 2015). Experimental evidence strongly suggests that in MS, OPC depletion is not a limiting step for remyelination but rather the inhibition of OPC recruitment and differentiation into myelinating oligodendrocytes (Boyd et al., 2013). In fact, a quantitative analysis of oligodendrocytes in MS lesions showed that only 30% of the lesions were devoid of OPCs, while the other 70% contain an increased number of OPC that were unable to differentiate and remyelinate axons (Lucchinetti et al., 1999). A recent study reported that rodent adult OPC have a transcriptome more similar to oligodendrocytes than to neonatal OPC. However, in demyelinating conditions, adult OPC are activated and revert their phenotype to a more neonatal OPC, and produce the cytokine IL-1β and chemokine CCL2 that enhance OPC mobilization and promote their repopulation in demyelinating areas (Moyon et al., 2015). This study shows that OPC can modulate neuroinflammation and promote regeneration and are not simple target cells.

#### Oligodendrocyte injury: cause or consequence of inflammation?

MS is an inflammatory disorder causing CNS demyelination and axonal injury. Although its etiology remains elusive, many reports associate autoimmunity, genetic predisposition, and environmental factors as triggers for MS pathogenesis. This autoimmune response is thought to be mediated by myelin-specific CD4^+^ T cells that initiate a series of neuroinflammatory events conjugated with those of either innate and adaptive infiltrating immune and CNS resident cells. This ultimately leads to the attack of myelin-producing oligodendrocytes, culminating in demyelination followed by axonal damage (Bauer et al., 2009; Nicol et al., 2015). In alternative to this long- favored hypothesis it has been proposed that the disease process could be triggered by events primarily occurring within the CNS. For example, it has been shown that primary oligodendroglial dystrophy in type III and IV lesions was followed by subsequent inflammation (Lucchinetti et al., 2000). Based on observations of lesions of patients with relapsing and remitting multiple sclerosis (RRMS) with extensive oligodendrocyte apoptosis, microglial activation, and few or no lymphocytes or myelin phagocytes (Barnett and Prineas, 2004), it has also been suggested a novel mechanism of new MS lesion formation initiation. This hypothesis is in agreement with the identification of extracellular myelin in MS leptomeninges and perivascular spaces, suggesting primary myelin trafficking from the CNS to the secondary lymphoid organs for further antigen presentation to immune cells to mount the autoimmune neuroinflammatory response (Fabriek et al., 2005; Kooi et al., 2009). Recently, using a transgenic mouse model for inducible depletion of adult oligodendrocytes it was verified that oligodendrocyte loss is followed by infiltration of CD4^+^ T cells into the CNS leading to a secondary, fatal demyelinating disease. Focal lesions with T cell infiltration and macrophage/microglia inflammation characterized this late onset demyelination. Moreover, myelin-specific T cells were identified in peripheral lymphoid organs and after isolation and *in vitro* activation could, by adoptive transfer, induce mild neurological symptoms, and inflammatory white matter lesions in the recipient animals (Traka et al., 2016). This study clearly raises the concept of autoimmunity to another level of complexity that requires a deeper understanding. Altogether, these data suggest that CNS demyelination is caused by a multitude of complex pathophysiologic mechanisms with several possible scenarios and cellular players. Therefore, it is extremely relevant to address when and how these interactions take place in de(re)myelinating conditions.

### Role of astrocytes in (re)myelination

#### Astrocyte phenotypes

Astrocytes (Andriezen, 1893) are originated from neural embryonic progenitor cells that line the lumen of the embryonic neural tube. However, they can be formed indirectly via radial glia, which in addition to function as scaffolding for newborn neuron migration, can serve as progenitor cells giving rise to astrocytes (Choi, 1981; Voigt, 1989; Kessaris et al., 2008). Astrocytic heterogeneity is far more complex than initially imagined and there is no complete consensus in their categorization. However, the classification of astrocytes by Ramón y Cajal into protoplasmic and fibrous astrocytes (Ramón Y Cajal, 1909) based on differences in their morphology, antigenic phenotype, location and function, is still valid and useful. Type 1 astrocytes (protoplasmic astrocytes) are localized in the gray matter and ensheath synapses and blood vessels to promote synapse and blood brain barrier functions, respectively. Type 2 astrocytes (fibrous astrocytes) are localized in the white matter and contact the nodes of Ranvier and the blood vessels (Barres, 2008; Sofroniew and Vinters, 2010). In addition, astrocytes can also be diverse in their ability to react in response to CNS insults. Astrocytes range from inactive or quiescent to active and reactive. Quiescent astrocytes exist in the normal resting CNS tissue. Upon injury or insult, astrocytes become activated by various mechanisms that result in mild astrogliosis. Reactive astrocytes are closer to the injury site and are responsible for the glial scar formation (Nash et al., 2011a). Astrocyte reactivity seems to influence myelination differently and it will be discussed in the next sections.

#### Astrocyte-derived promoters of oligodendrocyte proliferation, differentiation, and myelination

The processes by which astrocytes facilitate each step of myelination, including OPC proliferation, differentiation, initial oligodendrocyte-axon contact, and myelination, have been addressed in several studies (Figure 1). It is generally accepted that astrocytes support oligodendrocyte function. The first evidence of interplay between astrocytes and oligodendrocytes and its impact on myelination dates back to the middle 80's, when type 1 astrocytes were identified to expand O-2A progenitors from neonatal rat optic nerve. Such expansion was found to be mediated by unidentified soluble growth factors (Noble and Murray, 1984), later identified as platelet-derived growth factor (PDGF) (Noble et al., 1988; Richardson et al., 1988) and basic fibroblast growth factor (FGF2) (Bogler et al., 1990). PDGF and FGF2 are both potent mitogens for OPCs and inhibit premature oligodendrocyte differentiation.

Other soluble factors secreted by astrocytes have been implicated in enhancing myelination. Bhat and Pfeiffer observed that extracts from cultures enriched in astrocytes stimulated oligodendrocyte differentiation (Bhat and Pfeiffer, 1986), thus supporting the concept of a positive effect of astrocytes in myelination. In agreement with these findings, Gard and co-workers identified leukemia inhibitory factor-like protein (LIF) in conditioned medium produced by astrocytes that promoted oligodendrocyte survival and maintained them in a mature myelinogenic state (Gard et al., 1995). Ishibashi and colleagues also showed that astrocyte release of LIF in response to electrical activity in axons promoted oligodendrocyte myelination (Ishibashi et al., 2006). Other examples are neuregulin-1 (Taveggia et al., 2008), gamma-secretase (Watkins et al., 2008), ciliary neurotrophic factor (CNTF) (Stankoff et al., 2002), insulin-like growth factor 1 (IGF-1) (Ye et al., 2004; Zeger et al., 2007), osteopontin (Selvaraju et al., 2004), and neurotrophin-3 (NT3) (Kumar et al., 2007). Co-cultures of astrocytes and oligodendrocytes revealed that astrocyte proximity, in a contact-independent manner, induced profound changes in the levels of oligodendrocyte gene expression, in particular the expression of several myelin-related and cytokine receptors genes (Iacobas and Iacobas, 2010). Sorensen and colleagues showed that in cultures generated from rat embryonic spinal cord, neurosphere-derived astrocytes promoted myelination of CNS axons (Sorensen et al., 2008). Finally, Nash and colleagues found correlation of astrocyte phenotypes with their ability to support myelination. Activated astrocytes by CNTF increased myelination, while quiescent astrocytes induced by tenascin C through CXCL10 resulted in less myelinated fibers (Nash et al., 2011b). The analysis of postmortem brains of neonatal brain injury also revealed that the presence of activated STAT3 signaling in reactive astrocytes prevented the impairment of oligodendrocyte maturation (Nobuta et al., 2012).

Physical contact with astrocytes can also facilitate the maturation of oligodendrocytes (Sakurai et al., 1998). Astrocytes were found to promote adult mouse oligodendrocyte survival through a cell-contact dependent mechanism involving the interaction of α6β1 integrin on oligodendrocytes with laminin on astrocytes (Corley et al., 2001).

Moreover, myelin homeostasis in the CNS is dependent on the proper function of oligodendrocyte and astrocyte connexins (Orthmann-Murphy et al., 2008; Lutz et al., 2009). Astrocytes express connexin 43 (Cx43) and Cx30 and may couple to other astrocytes by homotypic gap junction composed of Cx43-Cx43 or Cx30-Cx30 while oligodendrocyte express Cx32 and Cx47 and also couple to one another using Cx32-Cx32 or Cx47-Cx47 gap junctions. Astrocytes and oligodendrocytes also establish heterotypic gap junctions composed of Cx47-Cx43 or, to a lesser extent, Cx32-Cx30 (Orthmann-Murphy et al., 2008). The elimination of gap junctions coupling oligodendrocytes and astrocytes in mice through oligodendrocytic Cx47 and astrocytic Cx30 delayed myelination, revealing a role for connexins and glial physical connection in myelin maintenance (Tress et al., 2012). The observation of a unidirectional flow through gap junctions, whereby cytosolic contents originating from astrocytes are preferentially transported to oligodendrocytes suggests that these cells are metabolically supported by astrocytes (Robinson et al., 1993). It was also proposed that astrocytes might buffer the concentration of potassium ions that accumulate in the white matter during neurotransmission in oligodendrocytes through gap junctions (Nagy and Rash, 2000).

In addition to promoting oligodendrocyte survival and differentiation, astrocytes also affect other aspects of oligodendrocyte biology. Astrocytes promote adult human oligodendrocyte process extension through FGF2 in combination with the ECM proteins fibronectin and laminin (Oh and Yong, 1996), in a protein kinase C (PKC)-dependent manner (Oh et al., 1997).

#### Astrocyte-derived promoters of remyelination

**Figure 2:**
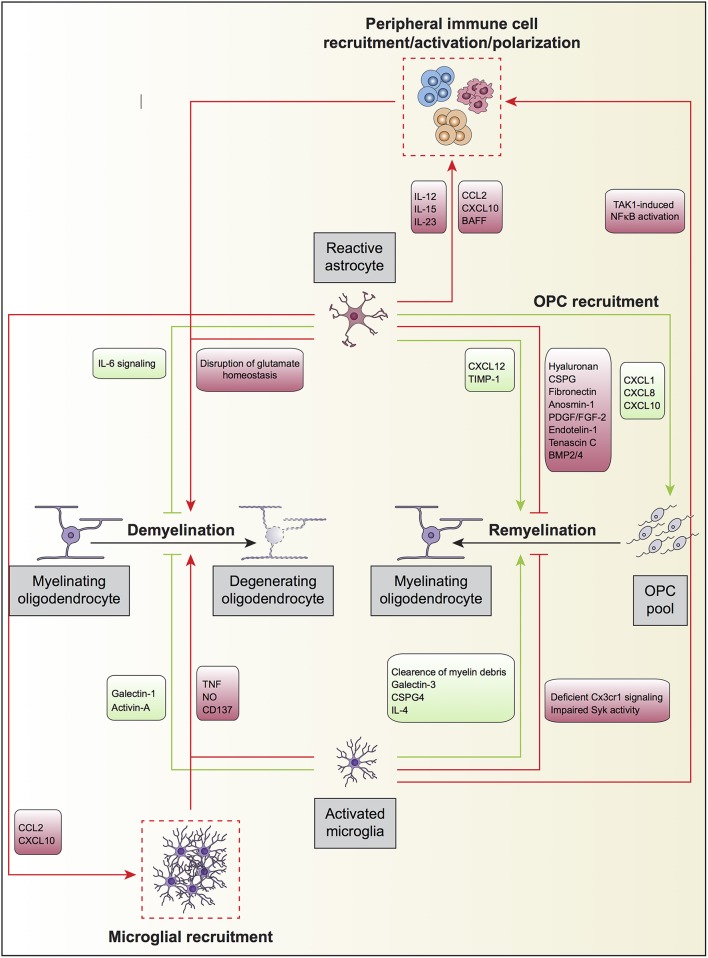
**Oligodendrocyte, astrocyte, and microglia crosstalk during demyelination and remyelination**. Upon an insult to the CNS parenchyma or in neurodegenerative diseases, mature myelinating oligodendrocytes degenerate and eventually die, a processes termed active demyelination. Reactive astrocytes and activated microglia directly participate in this process displaying both detrimental (red) and beneficial (green) roles. Astrocytes may also modulate the recruitment of peripheral immune cells by secreting different set of cytokines and chemokines, which will further promote the degeneration of myelinating oligodendrocytes. Furthermore, astrocytes secrete the chemokines CCL2 and CXCL10 to recruit overactive microglia, which may further increase oligodendrocyte loss. Microglial TAK1 signaling is also involved in the recruitment of peripheral immune cells to regulate active demyelination. On the other hand, astrocytes and microglia promote remyelination after myelin damage by the generation of oligodendrocytes from the OPC pool in the neuronal parenchyma. During remyelination, and as occurs for active demyelination, reactive astrocytes, and activated microglia can promote (green) or impede (red) the process. Astrocyte can further influence remyelination by secreting the chemokines CXCL1, CXCL8, and CXCL10 to recruit OPC to demyelinated zones where they can differentiate into mature oligodendrocytes.

In remyelination models using chemical demyelination, Franklin and colleagues showed that *in vivo* transplantation of type 1 astrocytes potentiated oligodendrocyte remyelination and increased the thickness of myelin sheaths (Franklin et al., 1991). In cuprizone-induced demyelination, the expression of TNFR2 in astrocytes resulted in the autocrine expression of CXCL12, which acted at its receptor CXCR4 on OPC, inducing their proliferation and differentiation, therefore enabling remyelination (Patel et al., 2012; Figure 2). In addition, CNTF was identified within activated/reactive astrocytes in and around spinal cord remyelinating lesions, and regulated FGF-2 production in astrocytes during early remyelination, suggesting CNTF as an important cytokine in demyelinating diseases (Albrecht et al., 2003). Skripuletz and co-workers showed that astrocyte ablation during cuprizone-induced demyelination did not prevent myelin damage but rather inhibited the removal of the myelin debris and delayed remyelination (Skripuletz et al., 2013). Interestingly, two studies using different models of demyelination of spinal cord white matter observed that the absence of astrocytes in the lesion area decreased the oligodendrocyte-mediated remyelination and increased remyelination mediated by Schwann cells (Talbott et al., 2005; Monteiro De Castro et al., 2015). Altogether, these results suggest that the astrocyte free regions of the lesion either contained inhibitory signals preventing terminal differentiation of OPC or lack appropriate signals necessary for OPC to undergo terminal differentiation.

#### Astrocyte-derived inhibitors of (re)myelination

Astrocytes have been initially described to have detrimental effects on oligodendrocyte differentiation, in particular those within the glial scar. Astrocytes within the glial scar inhibited regeneration and impacted negatively on remyelination (Fawcett and Asher, 1999; Silver and Miller, 2004). *In vitro* studies showed that type 1 astrocytes inhibited myelination of dorsal root ganglion axons by adult oligodendrocytes (Rosen et al., 1989). Astrocytes also secrete factors implicated in the inhibition of myelination and remyelination. Besides the above mentioned PDGF and FGF2 (Noble et al., 1988; Richardson et al., 1988; Bogler et al., 1990), which promote OPC proliferation and inhibit premature differentiation, others such as tenascin C (Nash et al., 2011b), bone morphogenetic proteins (BMP)2/4 (Wang et al., 2011), and hyaluronan (Sloane et al., 2010) have also been described. Hyaluronan is a glicosaminoglican (GAG) that interacts with CD44, a receptor present in OPC. This interaction impairs remyelination after lysolecithin-induced white matter demyelination because OPC do not differentiate into myelin-forming cells in demyelinating lesions where hyaluronan is present. Paralleling these findings, OPC differentiation was also blocked by treating OPC cultures with hyaluronan (Back et al., 2005). Astrocyte-derived endothelin-1 (ET-1) was identified to be a negative regulator of OPC differentiation and remyelination by promoting Jagged1 expression that activates Notch on OPC (Hammond et al., 2014). Blakemore and colleagues showed that remyelination was more efficient after transplantation of neonatal OPC into an astrocyte-free area of demyelination than into an area with established astrocytes (Blakemore et al., 2003). This contrasts with previous work from Franklin and colleagues, in which they showed a positive effect of astrocytes in remyelination, using a more acute model of demyelination (Franklin et al., 1991) (Figure 2). Finally, a recent report demonstrates that preventing astrocytic scar formation in SCI significantly reduces stimulation of axon regrowth (Anderson et al., 2016). Although no information concerning myelin was provided, this study supports a positive role for astrocytes during regeneration.

Overall, these studies suggest that the outcome of glial interactions in myelination is affected by the surrounding microenvironment, emphasizing that the activation state of astrocytes likely determines their permissive or inhibitory influence on oligodendrocyte development. Besides the different possibilities of astrocyte phenotypes, it should also be considered the distance of these populations to the lesion site, as relatively small changes in the responsive milieu may have different impacts on oligodendrocyte behavior. Nash and colleagues hypothesized that while astrocytes more distal to injury are activated cells that contribute in a greater extent to regeneration via the secretion of growth factors and cytokines, astrocytes in closer proximity to the lesion site are more reactive and may hinder the remyelination process (Nash et al., 2011a).

### Astrocytes in demyelinating diseases: focus on MS and EAE

Astrogliosis is one of MS pathological hallmarks. Astrocytes play an active role in both promoting demyelination and impairing remyelination by regulating peripheral immune cell trafficking, modulating BBB integrity, and being a source of chemokines and cytokines with pleiotropic functions (Figure 2).

#### Astrocytes and BBB function

BBB dysfunction is also a hallmark of MS progression. Astrocyte activation and loss of their end-feet around small blood vessels represent an early event in lesion development linked to BBB disruption in EAE (Correale and Farez, 2015). Astrocyte activation increases HIF-1, up-regulating VEGF-A expression, which induces the down-regulation of claudin-5 and occludin, important proteins that compose the tight junctions of endothelial cells, leading to BBB injury (Argaw et al., 2006, 2009, 2012). Gimenez and colleagues demonstrated that vascular adhesion molecule-1 (VCAM-1) expression, specifically by astrocytes, is crucial for T cell invasion of the CNS parenchyma in EAE (Gimenez et al., 2004). They also demonstrated that the VCAM-1 expression by astrocytes is dependent on TNFR1 expression (Gimenez et al., 2004). Still, it was observed in an *in vitro* BBB model (Megard et al., 2002), that IL-1β secretion by astrocytes is pivotal in mediating TNF-induced paracellular transport in endothelial cells (Didier et al., 2003).

#### Astrocytes and chemokine production/immune cell recruitment

Astrocytes secrete a plethora of chemokines and cytokines (Dong and Benveniste, 2001) that regulate different immune events during MS progression. Astrocytes were found to be the main source of the chemokine CCL2 in EAE (Ransohoff et al., 1993). This molecule is an essential chemoattractant for monocytes and T cells and exerts a key role in the onset of MS (Huang et al., 2001). Curiously, Toft-Hansen and colleagues, using transgenic mice expressing herpes simplex virus-derived thymidine kinase under the control of a glial fibrillary acidic protein promoter, investigated whether inhibition of reactive astrocytosis influences established EAE. In this experimental setting, the inhibition of reactive astrocytosis blocked the infiltration of T cells while the infiltration of monocytes were increased. It was also observed that the mRNA expression of CCL2 was upregulated, and of CXL10 downregulated, dissociating the reactive astrocytosis (GFAP expression) from CCL2 mRNA expression (Toft-Hansen et al., 2011). To corroborate the role of pro-inflammatory chemokines produced by astrocytes in MS pathology, Brambilla and colleagues blocked astroglial NF-κB activation in dominant negative transgenic mice (glial fibrillary acidic protein-IκBα). A clear reduction of chemokine gene expression was observed in these animals, which attenuated disease severity and improved functional recovery following EAE (Brambilla et al., 2009). In the same animal model, the authors also demonstrated a reduction of peripheral immune cell infiltration into the CNS at the chronic phase of EAE (Brambilla et al., 2014).

There is evidence that cytokines produced by astrocytes, such as IL-12, IL-23, and IL-15, regulate the myelin-specific auto-reactive response of effector T cells, namely by inducing the differentiation of CD4^+^ T cells in a pro-inflammatory phenotype such as Th1 or Th17 and also the cytotoxic activity of CD8^+^ T cells (Correale and Farez, 2015). Reducing astroglial NF-κB activation specifically attenuated the ability of T cells to produce pro-inflammatory cytokines during acute disease, suggesting that pro-inflammatory cytokines produced by astrocytes are important to regulate the ability of T cells to produce pro-inflammatory mediators (Brambilla et al., 2014). Th17 cells are major mediators of EAE progression and mice lacking IL-17 display less severe inflammation, indicating that IL-17-mediated signaling plays a critical role in the effector phase of EAE (Komiyama et al., 2006). The major function described for these cells is the coordination of local tissue inflammation through upregulation of pro-inflammatory cytokines and chemokines (Jovanovic et al., 1998). IL-17 acts through an heteromeric receptor complex, consisting of IL-17R (IL-17RA) and IL-17RC, which is expressed by a variety of cells including astrocytes (Gu et al., 2013). It was described that Act1 is an adaptor protein essential for the signaling mediated by IL-17 receptor (Chang et al., 2006) and induction of NF-κB activation (Li et al., 2000). Kang and colleagues demonstrated that the deletion of Act1 in the neuroectodermal lineage in mice results in attenuated EAE severity. Act1-deficient astrocytes showed impaired IL-17-mediated inflammatory gene induction (Kang et al., 2010), which was further corroborated by Yan and colleagues that knocked down Act1 expression specifically on astrocytes and effectively prevented EAE progression (Yan et al., 2012).

Astrocytes also regulate B cell activation by producing B cell–activating factor (BAFF), a cytokine that belongs to the TNF family. During MS, the production of this factor is increased in astrocytes in MS lesions (Krumbholz et al., 2005) and such increase may promote survival, expansion and activation of B cells during MS progression.

It was demonstrated that hypertrophic astrocytes produce the chemokines CCL2 and CXCL10 that activate microglia in the rim of the secondary progressive multiple sclerosis lesions (SPMS) with ongoing demyelination (Tanuma et al., 2006). As at this stage, leucocyte infiltration is at a minimum or absent, glial cells and glial-glial interaction might be the culprits sustaining demyelination. Using dominant-negative transgenic inhibition of astroglial NF-κB, it was observed that the number of total and activated microglial cells is reduced in chronic EAE. This resulted in a reduction of the overall inflammatory response and improved neurological function in GFAP-IκBα-dn mice (Brambilla et al., 2014).

#### Astrocytes acting on demyelination

In inflammatory and/or demyelinating conditions, astrocytes proliferate and may form glial scars composed of a dense network of hypertrophic cells. These reactive astrocytes have pronounced changes in the expression levels of adhesion molecules, antigen presentation molecules, cytokines, growth factors, receptors, enzymes, and protease inhibitors that modify the composition of the ECM.

Reactive astrocytes at the edge of active MS lesions express chemoattractant molecules for OPC, including CXCL8, CXCL1, and CXCL10, inducing their migration toward the demyelinated plaque, which might be important for remyelination (Omari et al., 2005).

It is well-recognized that the glial scar formation is crucial for helping restore blood brain barrier (BBB) integrity. However, in demyelinating conditions it also poses a physical barrier preventing OPC entry into the demyelinated area for interaction with denuded neurons (Fawcett and Asher, 1999; Silver and Miller, 2004; Nair et al., 2008; Wang et al., 2011). In the EAE model, it was observed that OPC could migrate toward the demyelinated lesion but were stuck at its margins, unable to penetrate the lesion site (Bannerman et al., 2007a; Williams et al., 2007). The glial scar not only represents a physical barrier but also a biochemical obstacle for remyelination. Astrocytes are capable of modifying the ECM in MS by secreting different components, which can directly affect remyelination in MS lesions (Clemente et al., 2013). For instance, astrocytes can produce a high molecular weight form of hyaluronan found to accumulate in chronic MS or EAE demyelinated lesions (Back et al., 2005). Moreover, chondroitin sulfate proteoglycans (CSPG) are produced by reactive astrocytes at the border of demyelinating areas (Lau et al., 2012). This ECM component inhibits OPC process outgrowth, differentiation and adhesion and impairs remyelination. CSPG-mediated remyelination impairment is dependent on protein tyrosine phosphatase sigma (PTPÏČ) and Rho-associated kinase (ROCK) activation (Pendleton et al., 2013). Inhibition of ROCK or RNAi-mediated down-regulation of PTPÏČ increases oligodendrocyte process outgrowth and myelination during exposure to CSPGs (Pendleton et al., 2013). Fibronectin is a glycoprotein of the ECM that inhibits the outgrowth of oligodendrocyte processes and myelin sheath formation (Siskova et al., 2009). Astrocytes secrete fibronectin in chronic MS lesions (Stoffels et al., 2013, 2015), stimulating OPC proliferation (Stoffels et al., 2015), but impairing oligodendrocyte differentiation and remyelination (Stoffels et al., 2013). Anosmin-1 is another astrocytic-secreted ECM associated glycoprotein that is deregulated in MS. Anosmin-1 is present at the core of chronic active and chronic inactive plaques (areas where remyelination is compromised). The presence of this component impedes OPC colonization of MS lesions and oligodendrocyte differentiation (Clemente et al., 2013). Reactive astrocytes over-secrete FGF-2 during EAE (Messersmith et al., 2000) and in MS plaques (Holley et al., 2003). This growth factor promotes OPC survival and proliferation but prevents their differentiation into mature oligodendrocyte (Goddard et al., 1999), impairing remyelination. Endothelin-1 (ET-1) is a secreted signaling peptide also highly expressed by reactive astrocytes in demyelinated lesions in MS (Hammond et al., 2014). This peptide reduces the rate of remyelination by acting indirectly in OPC and in an autocrine manner on astrocytes through endothelin-B receptor activation (Hammond et al., 2015).

Excitotoxic injury to the oligodendroglial lineage is another way by which astrocytes may impair remyelination during EAE. In normal conditions, astrocytes regulate glutamatergic neurotransmission by taking up extracellular glutamate from the CNS extracellular milieu and converting it into L-glutamine via activity of glutamine synthetase. During EAE, the expression of the glutamate transporters, GLAST, and GLT-1 (Ohgoh et al., 2002), glutamine synthetase and glutamate dehydrogenase (Hardin-Pouzet et al., 1997) are diminished in astrocytes, driving glutamate accumulation in the extracellular milieu, contributing to oligodendrocyte excitotoxic injury via activation of calcium-permeable AMPA receptors (Bannerman et al., 2007b).

Astrocyte-induced inflammation is harmful to myelin homeostasis and impairs remyelination. Inhibition of astroglial NF-κB during EAE, increases myelin preservation, improving myelin compaction, and remyelination (Brambilla et al., 2014). On the other hand, deletion of gp130, the signal-transducing receptor for cytokines of the IL-6 family, specifically in astrocytes during EAE leads to more severe disease. The loss of astrocytic gp130 expression resulted in apoptosis of astrocytes in inflammatory lesions, larger areas of demyelination, and increased numbers of CD4^+^ T cells within the CNS parenchyma (Haroon et al., 2011), demonstrating that the IL-6 signaling in astrocytes is important to reduce demyelination in EAE.

Another important aspect in glial-glial interaction during demyelination is the gap junction formation between astrocytes and oligodendrocytes (Lutz et al., 2009). During acute EAE, both Cx47 and Cx32 are severely reduced within and around lesions. The Cx47 protein was relocated intracellularly in oligodendrocytes, and its redistribution coincided with the loss of Cx43 in astrocytes (Markoullis et al., 2012). Moreover, there is loss of oligodendrocyte-oligodendrocyte and oligodendrocyte-astrocyte gap junctions and an increase in astrocyte-astrocyte gap junctions in gray matter MS lesions, suggesting that oligodendrocyte dissociation from reactive astrocytes may account for remyelination failure and disease progression in EAE (Markoullis et al., 2014).

On the other hand, metalloproteinase inhibitors expressed by astrocytes can play positive roles in oligodendrocyte response to injury. One known example is the tissue inhibitor of metalloproteinase (TIMP-1). In the EAE model, TIMP-1 is increased in reactive astrocytes, which regulates the secretion of matrix metalloproteinases from these cells (Nygardas and Hinkkanen, 2002). Moreover, TIMP-1 deficient mice exhibit poorer myelin repair in the EAE model (Crocker et al., 2006), have a specific deficit of NG2 positive OPC, and oligodendrogenesis is significantly impaired, correlating with dramatically reduced numbers of white matter astrocytes in the developing CNS (Moore et al., 2011).

### Modulation of (re)myelination and demyelination by microglia

Microglia constitute the myeloid resident population of the CNS, representing around 10% of the total glial cells within the nervous tissue (Soulet and Rivest, 2008). Microglia are critically involved in the scavenging of dying cells, pathogens, and molecules that engage pattern recognition receptors. As the major component of the immune effector system of the CNS at steady-state conditions, surveillant microglia are usually claimed to act as sensors of pathologic events (Hanisch and Kettenmann, 2007). Microglial activation has been described extensively in autoimmune diseases such as multiple sclerosis in humans and in the EAE mouse MS model. In these pathological contexts, microglia can produce and release neurotoxic (reactive oxygen and nitrogen species and glutamate) or neurotrophic molecules, pro and anti-inflammatory cytokines or chemokines, and present self-antigens to effector immune cells. Pathological evidence indicates that the remyelination onset in fresh lesions of brain and spinal cord of patients with MS occurs in acute, active lesions, which are characterized by a robust inflammatory response (Prineas et al., 1989). Following injury, different inflammatory molecules such as cytokines and chemokines are secreted and released by glial cells, inclusive microglia, in the surrounding milieu. In this scenario, microglia become activated, expand, migrate, and accumulate within the damaged area of the neuronal parenchyma, playing both beneficial and detrimental roles during myelin damage and repair (Figure 2).

#### Beneficial vs. detrimental roles of microglia in (re)myelination and demyelination

Although the literature relating microglia to myelination is not very extensive, several reports show that in homeostatic conditions microglia may also promote OPC survival and differentiation (Figure 1). Early studies using microglia and oligodendrocyte co-cultures showed that the former stimulated the synthesis of sulfatide, a myelin-specific galactolipid, as well as the expression of the myelin-specific proteins MBP and proteolipid protein (PLP) in oligodendrocytes, suggesting a positive role for microglia in myelination (Hamilton and Rome, 1994). In line with this, conditioned medium derived from non-activated microglia enhanced OPC survival and maturation through increase of PDGF-a receptor-signaling pathway and modulation of NF-kB activation (Nicholas et al., 2001). The same authors identified insulin-like growth factor-2 (IGF-2) to be an active promoter of oligodendrocyte survival, present in both conditioned media of non-activated and interferon gamma (IFNγ)-exposed microglia (Nicholas et al., 2002). Microglia can also promote OPC differentiation via galectin-3 but not galectin-1. Cultured OPCs exposed to conditioned medium from WT microglia had increased expression of MBP, while exposure of OPCs to conditioned medium obtained from galectin-3 deficient microglia resulted in decreased number of MPB^+^ cells (Pasquini et al., 2011). Galectin-3 expression on microglia has also been claimed to control OPC differentiation *in vivo* and to contribute to remyelination in a model of cuprizone induced-demyelination (Hoyos et al., 2014). On the other hand, galectin-1 biding to CD45 on the surface of microglia has been shown decrease their pro-inflammatory polarization, which attenuated myelin loss and neurodegeneration in the spinal cord of EAE mice (Starossom et al., 2012). Paralleling these findings, using the Cx3cr1 promoter to drive Cre recombinase expression only in microglia, it has been shown that the tyrosine TAK1 plays a crucial role in demyelination during EAE (Goldmann et al., 2013). Conditional ablation of microglial TAK1 largely attenuated myelin damage and disease severity in mice with EAE, an effect claimed to occur through defective recruitment of immune cell infiltrates, especially in the spinal cord (Goldmann et al., 2013).

The iron status of microglia is also important for oligodendrocyte survival. Increasing the iron load promotes the release of H-ferritin by microglia and incubation of oligodendrocyte cultures with conditioned medium of iron-loaded microglia increases the survival of these cultures (Zhang et al., 2006). In line with this, knocking down H-ferritin abrogated the trophic effect of the conditioned medium from iron-loaded microglia on oligodendrocyte cultures (Zhang et al., 2006).

Conditioned medium of non-activated microglia cultures was compared with that of astrocytes to evaluate their effect in OPC proliferation and differentiation. The results showed that astrocyte-conditioned medium was more efficient in promoting OPC proliferation, while microglia-conditioned medium accelerated oligodendrocyte differentiation more efficiently than that of astrocytes. Analysis of the media composition revealed that astrocyte-conditioned medium had increased levels of PDGF-AA, FGF2, FGF2 binding protein, CNTF, growth hormone, TIMP-1 and thrombospondin. In contrast, levels of IGF-1, E-selectin, fractalkine (CX3CL1), neuropilin-2, IL-2, IL-5, and vascular endothelial growth factor (VEGF) were significantly higher in microglia-conditioned medium. This distinct pattern of cytokines and growth factors in the conditioned medium of astrocytes and of microglia correlates with differentially activated intracellular signaling pathways in OPC exposed to the two different media (Pang et al., 2013).

In polarization conditions, such as *in vitro* stimulation with lipopolysaccharide (LPS), the release of cytotoxic effectors by both astrocytes and microglia produce the opposite effects on OPC (Pang et al., 2000). LPS-activated microglia hinders OPC differentiation by nitric oxide (NO)-dependent oxidative damage in an early phase and TNF in a later phase (Pang et al., 2010). In the presence of astrocytes, LPS-polarized microglia is toxic to differentiating oligodendrocytes via TNF signaling but not via NO-dependent oxidative damage (Li et al., 2008). Interestingly, the presence of astrocytes was claimed to quench the oxidative damage promoted by peroxynitrite, suggesting that TNF is the dominant mechanism in killing immature oligodendrocytes (Li et al., 2008). Oxidative stress, induced by CD137 ligand-polarized microglia, has also been demonstrated to promote oligodendrocyte apoptosis (Yeo et al., 2012). The effect of LPS-activated microglia was compared in OPC and mature oligodendrocytes *in vitro* and produced different outcomes. Pro-inflammatory microglia were found to reduce OPC viability (Miller et al., 2007) while alternatively activated microglia were shown to upregulate the GSK3β/AKT signaling pathway to enhance oligodendrocyte survival (Wang et al., 2015). In another report, LPS-activated microglia were shown to enhance the proliferation of Golli^+^ OPC in purified cultures (Filipovic and Zecevic, 2005) while Taylor and colleagues demonstrated that the conditioned medium of LPS-activated microglia decreased OPC proliferation globally (Taylor et al., 2010). Although LPS-polarized microglia increased the survival of mature oligodendrocytes in primary cultures (Miller et al., 2007), LPS activation of microglia in an *ex vivo* cerebellar organotypic culture model was shown to induce extensive demyelination via TNF production (Di Penta et al., 2013). As expected, the LPS effect in promoting oligodendrocyte toxicity is completely dependent on the expression of the LPS receptor TLR4 on microglia (Lehnardt et al., 2002). Furthermore, activated microglia accumulate in the early postnatal SVZ region and enhance oligodendrogenesis via released cytokines, such as TNF, IL-1β, IL-6, and IFNγ(Shigemoto-Mogami et al., 2014).

Microglia have also been shown to induce chemotaxis of OPC in culture. This effect is mediated by microglial secretion of hepatocyte growth factor when cultures are exposed to TGFβ (Lalive et al., 2005). IFNγ-exposed microglia can promote oligodendrogenesis; in this case, the microglial effect was highly correlated with IFNγ dosage, with high IFNγ doses preventing oligodendrocyte differentiation (Butovsky et al., 2006) and low IFNγ doses being supportive of oligodendrocyte generation (Butovsky et al., 2006). The effect of microglia polarized with high dose IFNγ could be reverted by exposing these cells to IL-4 (Butovsky et al., 2006). Besides, in chronic EAE, delivery of IL-4-polarized microglia to the CNS parenchyma, via injection into the third ventricle, could enhance oligodendrocyte differentiation and attenuate disease severity (Butovsky et al., 2006).

In remyelination, microglia have also been shown to play dual roles. Microglia expressing the CCR5 receptor were identified within early remyelinating lesions in patients at early stages of MS, suggesting a possible role for these cells in initiating remyelination (Trebst et al., 2008). Myelin repair is also influenced by microglia. Using the LysM promoter to ablate chondroitin sulfate proteoglycan 4 it has been shown that this molecule in microglia plays a significant role in promoting myelin repair after lysolecithin-induced demyelination (Kucharova and Stallcup, 2015). The phenotype of activated microglia was also shown to influence its beneficial role in efficient remyelination. Miron and colleagues found that the process of remyelination was dependent on microglia changing from an M1- to an M2-dominant response in the lysolecithin-induced demyelination model. Oligodendrocyte differentiation was enhanced with M2 microglia-conditioned medium *in vitro* and impaired *in vivo* following intra-lesional depletion of M2 microglia. Activin-A was identified to be one important mediator of the oligodendrocyte differentiation-promoting effect of M2 microglia (Miron et al., 2013). The M2 polarization of microglia induced by inhibiting class I/II histone deacetylases (Wang et al., 2015) or by treatment with Omega-3 polyunsaturated fatty acids (Chen et al., 2014), was also claimed to increase the preservation of myelin homeostasis after injury to the white matter caused by traumatic brain injury or cuprizone intoxication.

#### Microglia phagocytosis of myelin debris during myelin damage

Another important aspect of microglia in remyelination concerns its role in the clearance of myelin debris upon myelin injury. Myelin removal is a critical step in the remyelination process. To be effective, myelin debris must be cleared from the injury site (Kotter et al., 2006) and microglia have been recognized to play an active and beneficial role in this process (Neumann et al., 2009). Myelin clearance by microglia after cuprizone-induced demyelination was found to depend on the expression of microglial triggering receptor expressed on myeloid cells 2 (TREM2), a surface receptor that binds polyanions, such as dextran sulfate and bacterial LPS, and activates downstream signaling cascades through the adapter DAP12 (Poliani et al., 2015). This study showed that TREM2 is required for promoting microglial expansion in response to myelin damage. Moreover, a subpopulation of microglia was identified to be responsible for producing IFNβ in demyelinated lesions of EAE, a commonly used cytokine to treat RRMS. IFNβ-producing microglia are closely associated with myelin debris in the injured CNS at the peak of EAE and treatment of naïve microglia with IFNβ improved removal of myelin debris in demyelinated organotypic cultures (Kocur et al., 2015). In the cuprizone model, astrocytes are thought to recruit microglia to the lesion site in order to phagocyte and clear damaged myelin, a process regulated by the chemokine CXCL10. In the absence of astrocytes and, consequently, microglia recruitment, removal of myelin debris is significantly delayed inhibiting OPC proliferation and remyelination (Skripuletz et al., 2013). In line with this, it has been shown that the clearance of myelin debris by microglia can be blocked in Cx3cr1-deficient mice, which have a clear deficit in microglial phagocytosis and exhibit persistent myelin deficits that correlate with a deficiency in OPC recruitment. On the other hand, the tyrosine kinase Syk has been shown to sensitize cultured microglia to phagocyte myelin (Hadas et al., 2012). These data reinforce the important role microglia play in myelin clearance and their impact in proper remyelination (Lampron et al., 2015). Myelin can also down-regulate its own phagocytosis by microglia through CD47-SIRPa, suggesting that the same mechanism that protects myelin as “self” antigen from phagocytosis may turn disadvantageous when clearance of degenerated myelin is necessary (Gitik et al., 2011).

## Concluding remarks

Homeostasis of the CNS myelination depends on the crosstalk between oligodendrocytes, astrocytes, and microglia. Understanding the nature and complex dynamics of such interactions, which can play both beneficial and detrimental roles during damage and repair, will increase our knowledge into demyelinating diseases, and may help us to devise novel and more holistic ways to manipulate and improve remyelination.

## Author contributions

HD, CP, and RS contributed equally to this work.

## Funding

HD, CP and RS are supported with fellowships funded by Fundação para a Ciência e Tecnologia (FCT) (references SFRH/BPD/90268/2012 to HD, SFRH/BPD/91962/2012 to CP and SFRH/BPD/91833/2012 to RS).

### Conflict of interest statement

The authors declare that the research was conducted in the absence of any commercial or financial relationships that could be construed as a potential conflict of interest.
