# Corrigendum: Oligodendrocyte, Astrocyte and Microglia Crosstalk in Myelin Development, Damage, and Repair

**DOI:** 10.3389/fcell.2016.00079

**Published:** 2016-07-19

**Authors:** Helena S. Domingues, Camila C. Portugal, Renato Socodato, João B. Relvas

**Affiliations:** ^1^Glial Cell Biology Group, Instituto de Biologia Molecular e Celular, Universidade do PortoPorto, Portugal; ^2^Glial Cell Biology Group, Instituto de Investigação e Inovação em Saúde, Universidade do PortoPorto, Portugal

**Keywords:** oligodendrocyte, astrocyte, microglia, multiple sclerosis (MS), experimental autoimmune encephalomyelitis (EAE), myelination, demyelination, remyelination

Reason for Corrigendum:

In the original Review article, we neglected to thank some of our sponsors. The correct funding statement is shown below. The authors apologize for this oversight. This error does not change the scientific conclusions of the article in any way.

Moreover, the Editorial Office and the handling Editor, Adelaide Fernandes, would like to state that while she published with Helena Sofia Domingues and João Bettencourt Relvas in 2014 and 2015, the review of the article was handled objectively.

## Funding

HD, CP, and RS are supported with fellowships funded by Fundação para a Ciência e Tecnologia (FCT) (references SFRH/BPD/90268/2012 to HD, SFRH/BPD/91962/2012 to CP and SFRH/BPD/91833/2012 to RS). This article was funded by the project Norte-01-0145-FEDER-000008000008—Porto Neurosciences and Neurologic Disease Research Initiative at I3S, supported by Norte Portugal Regional Operational Programme (NORTE 2020), under the PORTUGAL 2020 Partnership Agreement, through the European Regional Development Fund (ERDF).

### Conflict of interest statement

The handling Editor has declared a past co-authorship with the authors HD and JR and states that the process nevertheless met the standards of a fair and objective review. The other authors declare that the research was conducted in the absence of any commercial or financial relationships that could be construed as a potential conflict of interest.

